# On Febrile Origin

**Published:** 1818-05

**Authors:** 


*For the London Medical and Physical Journal.*

*On Febrile Origin;* by Dr. KINGLAKE.

THE origin of febrile disease is unquestionably very various, and is often so obscured by incidental and collateral circumstances, as to render a correct discrimination of its source extremely difficult. But a well-founded distinction may be justly made, as to whether the disease has a local or general commencement. The universality of febrile action is such as to render it inconceivable that such an extent and violence of disease could have at once arisen, and have been manifested compatibly with the continuance of life. A disorder so general, and so sudden, would, it may be presumed, involve the vital functions of the animal economy in such a state of embarrassment as would necessarily, *ipso facto*, be destructive to existence. Deviations from the healthy state, of whatever nature or degree, are, for the most part, slow in their approach, and gradual in their progress. Seasonable and ample warning is given of the onset of diseases, by the disturbed sensations arising from the deranged actions and functions of the various parts of the system. The salutary actions of life are bound and linked together by the mutual ties of organic sympathies. The circumstance of one part being in a state of health is an efficient law in the animal economy for other parts to be in a similar condition; and thus a reciprocity of vital and healthful influence subsists between both the contiguous and remote parts of the animal frame. But for this innate connexion, there would be neither entireness nor harmony in the various and dissimilar functions of animal life. The beneficence of Nature has happily provided, in the difference of structure of the animal frame, barriers or obstacles to the easy transmission of disordered action. It is not every slight departure from the healthy state that either establishes or propagates itself. The part that may be its seat re-acts on the morbid intrusion, and at least restrains its threatened mischief, if it should not overcome and annul it. The conflict on these occasions announces itself in such a way as to render the attack an object of seasonable attention. If the resisting or counteracting power in the diseased part should be unequal to its own defence, and should give way to the morbid circumstances by which it might have been assailed, the system, more or less generally, and with greater or less violence, will partake of the commotion, until that which at first occupied, as it were, but a part, may be at length gradually extended over the whole frame. It is in this way, it may be presumed, that all febrile diseases, which are commonly regarded as of a general nature, originate, and finally become diffused over the system, agreeably to the natural laws of associative or sympathetic action.

It is obvious that febrile affections will, in their radical, as well as external, character, be considerably modified by the properties of the agents by which they may be induced. It is also equally evident that constitution, habit, climate, and other physical and moral circumstances, will exert their respective and peculiar influence in producing the description of fever that might exist. The external character of fever may be almost infinitely diversified by various causes; but the intrinsic nature of the febrile state admits of the important practical generalization, that all the forms of that disease are of a local origin, and are never, in their onset, commensurate with the whole system. The doctrine of fever is much simplified by this view of its fundamental nature, and cannot fail usefully to point out, as the leading object of inquiry, what particular part might have been the seat of the disease that has been sympathetically extended so far over the system as to assume the form of febrile affection.

The true nature of fever will be much elucidated by looking to its probable source and dissemination, instead of embarrassing the attention with unmeaning calculation as to occult influences, and supinely watching the course of the general conflict, without efficiently relieving the local disturbance from which the extended mischief had originated. The importance of the distinction here insisted on refers rather to low remittent fevers, usually considered as of the typhoid kind, in which the attendant symptoms do not directly suggest the existence of visceral disease; or, at least, not in a degree sufficient to have given birth to the affection. Instead of a vital organ being suspected to be the source of such diseases, when any particular viscus is especially affected, it is held to have been incidental and secondary, rather than essential and original. When the commencement and progress of visceral disease are such as denote active inflammation, no doubt remains on the subject, and the treatment is solely directed to remedy the local ailment. The arbitrary distinction which has been raised between inflammatory and febrile diseases has been productive of much mischief: they probably never differ but in the *majus* and *minus*; they are radically the same, and to dispute the identity of principle from whence their different forms proceed, would be to multiply words without distinguishing things. It does not at all follow, that, because a vital organ is not agitated with the more violent pains of inflammatory action, that it should not still be disordered by a state of excitement that may be subversive of its healthy function, from whence may arise morbid sympathies, that may be distributed over the whole frame. The symptoms of this state may not be evidently inflammatory, but the suffering viscus has nevertheless to cope with congestive fullness and painful action of vessel, that constitute a very serious state of disease, and will require, for its relief and cure, a watchful attention to its local nature, rather than an unavailing endeavour to alleviate the symptoms, without such due reference to the morbid source from whence they proceed.

Agreeably to the view which has here been taken of the febrile state of disease, it is clear that it is a morbid condition that implies a general affection of the system, resulting from some local derangement of the healthy condition of some part of the frame. The state of fever, therefore, although of a secondary nature, implies a condition of disease always serious from the magnitude of its extent, and as possessing a degree of danger proportioned to the vital importance of the part from which it derives its origin. The brain is the grand source of sensation; its healthful state cannot be disordered without, in some measure, distempering the sensibility, and associatively disturbing the various functions of the whole frame. It is not uncommon for the brain to be oppressed by vascular fullness, in a degree not only to disorder its healthy sensation, but also to draw into sympathy with it the heart, stomach, intestines, lungs, liver, kidneys, and other parts, inducing the full form and character of febrile disease. This affection may run on to an indefinite period,—at least, until either destructive exhaustion, disorganization, effusion, or extravasation, ensue; or the re-active powers of the system may ultimately restore the healthy state of the brain, when the sympathetic part of the malady would subside, and terminate in health.

The sensitive nature of the brain, and the various and complicated functions which it has to perform in the animal economy, render it peculiarly susceptible of becoming affected by slight causes of disease. Occurrences that would be harmless in any other viscus, might be seriously injurious to the brain. The affection may not, indeed, be inflammatory: the morbid excitement may not go to that extent; yet it deranges the healthy function, and thereby disturbs the whole system sufficiently to induce the ordinary appearance of febrile disease. The leading inquiry in all febrile diseases should be, to ascertain what vital organ is most affected; and the history of the case should be sufficiently investigated, to determine whether the suffering organ was primarily or secondarily affected. The danger of fever is generally to be estimated by the importance of the vital organ that may have originated it. When it occurs on the brain, the hazard is most formidable; the heart and stomach are also organs of fearful disease. The lungs, liver, kidneys, &c. are attended with proportionate risk and uncertainty. If fevers of every kind are radically similar in being of local origin, it is clear that the indication of cure will partake of that radical resemblance. From the excessive tone and action emanating from inflammatory excitement, and occurring in either the visceral, muscular, membranous, ligamentous, or any other kind of structure, down to the less-developed conditions of morbid action, presenting in the low remittent kind of fever, it becomes equally necessary that the burthen of disease should be removed from the affected part, seeing that on its cessation depends the subsiding of the general affection.

Vascular depletion, and intestinal evacuation, to an extent that would be likely to disembarrass the affected parts, will be required; and, although a tardy convalescence may be expedited by tonic and stimulant treatment, yet, pending febrile affection, it is probable that the local cause of the general ailment still exists, and that, until it be effectually relieved, the feverish state will not be overcome. As long as the local origin of the disease exists, its extension, of course, will remain; and, during that time, stimulating agents must add to the diseased action, and tend to incur either destructive exhaustion, or irretrievable disorganization. The abstraction of morbid stimuli is the grand object to be pursued in the cure of all diseases dependent on either vitiated, inordinate, or excessive action. These morbid stimuli are commonly excessive arterial action, undue vascular distension, unequal distribution of the circulating fluids, irregular and distempered secretions, inactive bowels, thirst, deficient perspiration, intemperate heat, mental anxiety, &c. These should be either moderated or wholly withheld, according to the exigencies of the case, when the order and calmness of healthy action would, most likely, be resumed and established. An increased action of the heart and arteries, with deranged secretion, denote an active source of disease, which must be overcome by means of abstraction, rather than of supply; or, to speak more medically, by sedative rather than by stimulant influence, before the healthful state can be restored. But, when the powers of life begin to flag, and the evidence of immoderate excitement is wanting, it will be requisite to sustain the drooping state of vital energy in such a way as may afford a chance of restoring it to the healthy equilibrium. Pure air, adequate warmth, aliment, sleep, and mental tranquillity, are the most congenial and salutary stimulants on these occasions; and, when the local ailment has not mortally injured the affected part, recovery may be calculated on as the natural and reasonable result of such agency.

*Taunton; March* 12, 1818.

